# Very rapid long-distance sea crossing by a migratory bird

**DOI:** 10.1038/srep38154

**Published:** 2016-11-30

**Authors:** José A. Alves, Maria P. Dias, Verónica Méndez, Borgný Katrínardóttir, Tómas G. Gunnarsson

**Affiliations:** 1 DBIO & CESAM-Centre for Environmental and Marine Studies, University of Aveiro, Aveiro, Portugal; 2 South Iceland Research Centre, University of Iceland, Fjolheimar IS-800 Selfoss & IS-861 Gunnarsholt, Iceland; 3 Birdife International, The David Attenborough Building, Pembroke Street, Cambridge, CB2 3QZ, UK; 4 MARE-Marine and Environmental Sciences Centre, ISPA-Instituto Universitário, Rua Jardim do Tabaco 34, 1149-041, Lisboa, Portugal; 5 School of Biological Sciences, University of East Anglia, Norwich Research Park, Norwich, NR4 7TJ, UK; 6 Ecology Department, Icelandic Institute of Natural History, 210, Gardabaer, Iceland

## Abstract

Landbirds undertaking within-continent migrations have the possibility to stop *en route*, but most long-distance migrants must also undertake large non-stop sea crossings, the length of which can vary greatly. For shorebirds migrating from Iceland to West Africa, the shortest route would involve one of the longest continuous sea crossings while alternative, mostly overland, routes are available. Using geolocators to track the migration of Icelandic whimbrels (*Numenius phaeopus)*, we show that they can complete a round-trip of 11,000 km making two non-stop sea crossings and flying at speeds of up to 24 m s^−1^; the fastest recorded for shorebirds flying over the ocean. Although wind support could reduce flight energetic costs, whimbrels faced headwinds up to twice their ground speed, indicating that unfavourable and potentially fatal weather conditions are not uncommon. Such apparently high risk migrations might be more common than previously thought, with potential fitness gains outweighing the costs.

Recent advances in tracking movements of individual birds are revolutionising our understanding of avian migration^1^,^2^. New tracking technologies (e.g. geolocators and PTT transmitters) have revealed migratory journeys in excess of 5000 km of active flight^1^,^2^,^3^, setting the endurance exercise record of any animal^4^. Ultra long-distance continuous migratory flights have been suggested to facilitate avoidance of predators, parasites and pathogens^5^, but such continuous exercise is also known to increase mortality risk^4^. Migratory landbirds typically avoid crossing large ecological barriers such as mountain-ranges, deserts and oceans^6^,^7^, often using routes over suitable habitats along which stopping to rest and refuel is possible, for example by following the edge of continental land-masses^6^. However, when detours from the shortest route considerably increase travel distance, energy or time, crossing ecological barriers may be beneficial^1^. Birds breeding at high latitudes are often those that undertake the longest non-stop flights, crossing oceans^1^,^8^,^9^,^10^, ice-caps^11^ and deserts^2^, but such long flights can incur high mortality risk due to exhaustion associated with prolonged unfavourable weather conditions^6^. Species migrating by flying long distances continuously over oceanic waters^1^,^3^,^8^,^12^ and land masses^2^ all undertake stop-overs during either both journeys^2^,^3^,^8^,^12^ or pre-nuptial migration only^1^,^6^. During pre-nuptial migration, the use of stop-over sites might allow gauging conditions closer to the Arctic and subarctic breeding grounds as arriving too early can also be disadvantageous if conditions are unsuitable upon arrival^6^. Additionally, by refuelling during migration, birds can restore body reserves thus increasing the likelihood of reaching the breeding grounds in good condition and at the right time^6^,^13^. Non-stop long distance flights over unsuitable habitats entail considerable survival risk as no sheltering options are available and future weather conditions in distant locations are impossible to predict^14^. Indeed the largest mortality events recorded on migration (5000–200 000 individuals^15^,^16^,^17^,^18^) refer to landbirds crossing large waterbodies and encountering adverse weather conditions^6^. However, favourable winds can also play a fundamental role in the crossing over large expanses of unsuitable habitat such as oceans^6^. Many species wait for tailwinds at coastal sites for several days before embarking on large sea crossings, departing only when wind subsidies are considerable^6^,^14^. If significant headwinds are encountered during oceanic flights, birds must endure substantially longer flight periods which can result in depletion of fat and muscles and exhaustion^19^. It is therefore expected that birds depart for long distance sea crossings with favourable tailwinds and that flight air speeds are as high as possible, independent of winds encountered *en route*, in order to reduce exposure to potentially adverse conditions.

Iceland hosts important populations of several migratory wader (or shorebird) species^20^ which winter in Europe and west Africa^21^,^22^. These species have to negotiate one of the longest continuous sea crossing of all Arctic and subarctic breeding landbirds^6^. Species migrating to west Africa (e.g. the Bijagós archipelago in Guinea-Bissau) could either undertake a non-stop oceanic flight (~5800 km) or, after an initial sea crossing (~800 km) to the UK, follow the continental land masses to the wintering grounds (~5200 km). Both alternatives result in similar distances and some Icelandic breeding waders are known to follow the coastline^21^, even those for which a single flight overwater is potentially feasible^23^. The extent to which either of these routes is used is not known and although the non-stop oceanic flight is potentially of higher risk, this will likely depend on wind conditions during migration. We deployed geolocator tags on Icelandic whimbrels (*Numenius phaeopus islandicus* Brehm, 1831), a species which is known to winter in West Africa^22^ to investigate (1) if non-stop flights over oceanic waters between Iceland and West Africa are undertaken during autumn and spring migration; (2) the level of wind support encountered *en route* and how this affects flight speed.

## Results and Discussion

**Figure 1:**
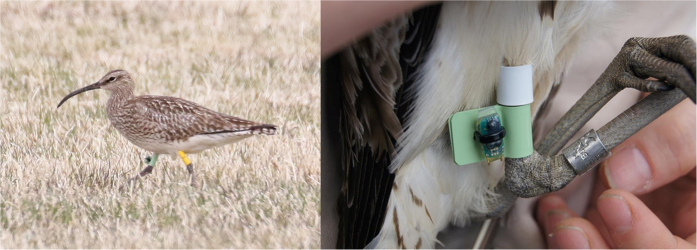
Icelandic whimbrel (*Numenius phaeopus islandicus*) carrying a geolocator (left: photo by Tómas G. Gunnarsson) attached to a leg flag (right: photo by Camilo Carneiro).

**Figure 2:**
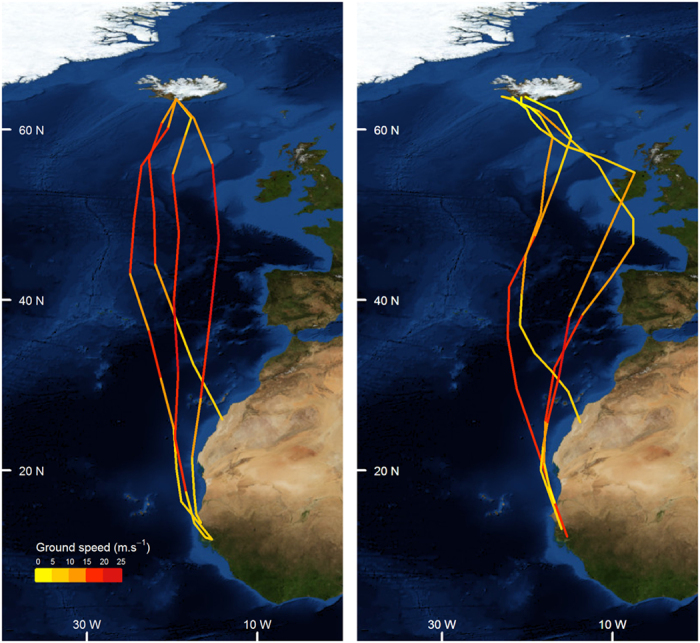
Geotracked migratory routes of four Icelandic whimbrels (*Numenius phaeopus islandicus*) between breeding sites in Iceland and wintering areas in West Africa during post-nuptial/autumn (left) and pre-nuptial/spring (right) migration. Track sections are coloured as a function of ground speed. All individuals flew non-stop in Autumn whilst two made a stop-over in the UK or Ireland in Spring (details in Table 1). Maps created using R 3.1.2 using packages ggplot2, ggmap, raster and RgoogleMaps^33^ (image data providers: US Dept. of State Geographer © 2016 Google) in WSG 48 coordinate reference system.

Ten adult breeding Icelandic whimbrels were tagged with geolocators in June 2012 (Fig. 1), seven were recorded on their territories in June 2013, of which four tags with data were retrieved. During the post-nuptial migration in autumn 2012, all four whimbrels flew non-stop to their wintering areas in west Africa (Fig. 2), covering distances of ~3900 to 5500 km in 5 days (Table 1) and, on occasion, achieving the fastest recorded speeds for terrestrial birds on long-distance flight over oceanic waters (up to 18–24 m s^−1^). During the return migration, two of these birds stopped for 11 (Male 2) and 15 days (Female 2), covering a total distance of ~10,500 and 11,000 km, respectively. However, the remaining female and male completed the return migration in another continuous flight (Fig. 2) for a total round trip of ~7800 (Female 1) and 11,000 (Male 1) km, respectively (Table 1). Completing such a long annual migration cycle in two long-distance flights is highly unusual for species where such studies have been undertaken. Only one other long-distance Arctic migrant wader, the Pacific Golden Plover (*Pluvialis fulva*), has been recorded undertaking such non-stop flights (ca. 9700 km total distance) between Alaska and Hawaii^9^. For this species however, no alternative route over coastal land masses is possible without considerably increasing flight distance.

**Figure 3:**
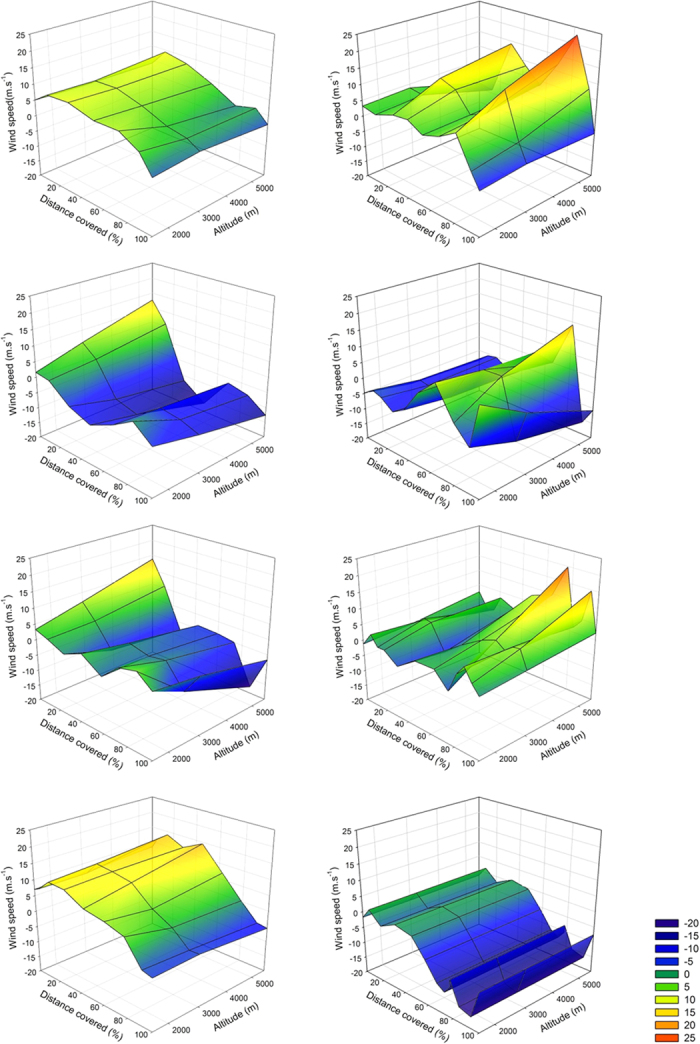
Variation in wind speed (m s^−1^) experienced during autumn (left column) and spring (right column) migratory flights of four Icelandic whimbrels. Each row is one individual, following Table 1, from top to bottom: Male 1, Female 1, Male 2, Female 2. Negative wind values (blue) indicate headwind and positive values indicate tailwind. Wind was estimated for three flight altitudes: 1500, 3000 and 5500 m. Distance from departure location was converted into percentage for ease of interpretation.

In order to assess how wind conditions encountered on these journeys varied, particularly for flights with and without stopovers, we quantified wind support at each position just prior to and during the migratory flight. All four whimbrels departed Iceland in favourable wind conditions (i.e. tailwinds), but all four arrived in West Africa having faced headwinds, mostly in the later part of the journey (Fig. 3). Conversely, only one individual departed from the winter grounds in favourable wind conditions (Male 1, the earliest to depart), while all others departed in headwinds of 1.3–4.8 m s^−1^, with one individual encountering headwinds for virtually the entire journey, including after stopping-over (Female 2, Fig. 3). A pre-nuptial migration strategy involving a stopover is likely safer regarding potentially unfavourable weather conditions encountered *en route* and upon arrival, but stopping to refuel will reduce overall migration speed. Indeed, and despite all tracked individuals departing the wintering grounds at similar times (22^nd^–29^th^ of April), those flying non-stop arrived before those that undertook a stopover. This includes the last individual to depart which flew non-stop (Female 1) and arrived at the breeding grounds 6 to 10 days before the two individuals that made a stopover (of 11–15 days), thus overtaking its conspecifics^23^. The earlier arrival of the female and male that flew non-stop to Iceland could be advantageous if they capitalize by nesting early, as this is known to increase breeding success, particularly in Arctic and subarctic systems^24^. However, laying dates did not differ substantially between the individuals undertaking direct flights (20^th^–31^st^ of May) or those that made a stopover (25^th^ May–7^th^ of June), suggesting that timing of breeding is constrained by other or additional factors, such as environmental conditions for nesting or timing of mate arrival (Supplementary Fig. S1).

The very fast ground speeds achieved by migrating whimbrels were influenced by the wind speeds encountered *en route*, particularly at altitudes of 1500 m (Table 2). Wind support at this altitude accounts for 4 to 36% of fastest speeds of each individual, with the highest wind assistance corresponding to the maximum recorded ground speed of 24.2 m s^−1^ (87 km h^−1^). Some individuals also reach very fasts speeds whilst facing headwinds which can be 2 to 40% of their ground speed, and in its most extreme case resulting in airspeed of 25.0 m s^−1^ (90 km h^−1^; Female 2). Average speeds for the entire continuous migratory flight are similar to those of other species crossing oceans (50–65 km h^−1 ^5,8,9) which are also strongly influenced by wind speed *en route*^5^. By flying at high speeds and non-stop over open ocean these species reduce the time on migration and might be using an “airspace corridor” to avoid predators, parasites and pathogens^5^. But if wind conditions at distant locations along the route are unfavourable, such migratory strategy can result in mortality by exhaustion, even after arriving at destination (TGG, pers. observation).

Our ability to track migration is providing new insights into the extraordinary capacity of birds to move extremely fast over very large distances, by continuously sustaining endurance exercise during several days. Wind support is crucial during such extreme journeys, but current predictions of changes in climatic patterns, specifically changes on regional scale wind patterns^25^, can potentially have a considerably disproportionate negative effect on those species that regularly undertake non-stop long distance flights over unsuitable habitats. Variation in migratory strategies within the same population will likely allow coping with potential changes, but predicting such responses requires an understanding on how these migration strategies can arise and are maintained. In addition, by linking different migratory strategies to associated fitness consequences will be key in our ability to anticipate demographic changes for migratory populations.

## Methods

### Bird tracking

Given Icelandic whimbrels previously established adult return rate (ca. 60–80%)^26^, in June 2012 we deployed 10 geolocators (Intigeo W65A9RJ, Migrate Technology) on breeding birds in South Iceland (63° 47N, 20° 12W). All individuals were caught with a nest trap (Moudry TR60; www.moudry.cz), ringed with metal and colour rings and measured to determine sex^26^. Seven of these birds were recorded breeding in the same location 12 months later, five of which (two females and three males) were re-captured and the geolocator collected. All of these individuals were from different pairs and the geolocator of one male was corrupted as a result of saltwater entering the device, leaving four individuals available for analysis. All animal handling and protocols were carried out in accordance with relevant guidelines and regulations under licenses issued by Icelandic (Natural History Institute; license number 365) and International regulatory bodies (International Wader Study Group; license number 1235).

### Positional data and flight speed

Light data from the geolocators were smoothed twice^27^ and used to estimate positions^28^ during migration (i.e. between Iceland and West Africa) using IntiProc (v. 1.03, Migrate Technology, Ltd.) and “GeoLight” package in R^29^, assuming a sun elevation angle of −6° based on *in situ* geolocator calibration prior to deployment. Total migration length, distance (great circle route) and speed were estimated between the last and first positions on land in the breeding areas (Iceland) or wintering areas (W Africa). As geolocator positions are only attained at a minimum of 12 hours intervals, flight speed (time taken to cover the distance between two sequential positions-in m s^−1^) was estimated for each 12 hour flight segment defined as two sequential positions between the first location outside the breeding, wintering or stop-over areas and the first location on land (at breeding, wintering or stop-over areas).

### Wind support

Wind data at the location (±2.5 degrees) and time of each geolocator recorded position was extracted from NOAA using the dataset Reanalysis by NCEP^30^. Headwind or tailwind vector between sequential positions was interpolated for three altitudes (1500, 3000 and 5500 m) using function “NCEP.interp” from package RNCEP^31^. To test at which altitude wind speed had an effect on ground speed, we built a GLMM with individual as random factor to control the non-independence and DFs were calculated using the Satterthwaite approximation in package lmerTest. All analysis and calculations were performed in R 2.15.0^32^.

## Additional Information

**How to cite this article**: Alves, J. A. *et al*. Very rapid long-distance sea crossing by a migratory bird. *Sci. Rep.* **6**, 38154; doi: 10.1038/srep38154 (2016).

**Publisher's note:** Springer Nature remains neutral with regard to jurisdictional claims in published maps and institutional affiliations.

## Supplementary Material

Supplementary Information

## Figures and Tables

**Table 1:** Timings, distances and speed of total migration and non-stop flights by Icelandic whimbrels that flew direct to the winter grounds (all birds in Autumn) and that made a stop-over (Male 2 and Female 2) or a direct flight (Male 1 and Female 1) in the return migration (Spring).

Migration	Atumn				Spring			
	Male 1	Male 2	Female 1	Female 2	Male 1	Male 2	Female 1	Female 2
Onset of migration (departure)	03-Aug	06-Aug	06-Aug	03-Aug	20-Apr	22-Apr	29-Apr	23-Apr
End of migration (arrival)	07-Aug	10-Aug	10-Aug	07-Aug	25-Apr	10-May	04-May	14-May
Total duration (d)	5	5	5	5	6	19	6	22
Stopover time (d)	0	0	0	0	0	11	0	15
Total migration distance (km)	5425	5171	3898	5535	5555	5364	3865	5560
Total duration (h)	107.8	107.9	78.9	120.0	121.2	444.1	127.3	540.6
Total migration speed (km h^−1^)	50.31	47.94	49.38	46.14	45.83	12.08	30.37	10.29
Non-stop flights (over ground speed)								
Max speed (m s^−1^)	24.18	18.60	17.93	21.91	19.71	21.29	13.45	18.19
Min speed (m s^−1^)	5.75	9.89	5.66	5.36	3.57	2.19	3.08	4.79
Average (m s^−1^)	15.55	14.38	13.32	14.35	13.87	8.24	9.09	9.63
Sd	6.84	3.00	4.19	6.42	5.10	5.40	3.17	4.25
N	7	8	8	9	10	14	11	15

**Table 2:** Results of GLMM of wind speed at three altitudes on ground speed of four Icelandic whimbrels tracked during migration between Iceland and West Africa.

	Estimate (SE)	df	t value	p value
Intercept	12.11 (0.56)	71	21.487	<0.001
Wind 1500 m	0.51 (0.21)	71	2.436	0.017
Wind 3000 m	0.16 (0.29)	71	−1.197	0.235
Wind 5500 m	0.70 (0.13)	71	1.433	0.156
