# Corrigendum: Very rapid long-distance sea crossing by a migratory bird

**DOI:** 10.1038/srep44631

**Published:** 2017-04-28

**Authors:** José A. Alves, Maria P. Dias, Verónica Méndez, Borgný Katrínardóttir, Tómas G. Gunnarsson

Scientific Reports 6: Article number: 3815410.1038/srep38154; published online: 11 30 2016; updated: 04 28 2017

The original version of this Article contained an error. The authors reported the maximum flight speed of Whimbrels (*Numenius phaeopus*) as the fastest recorded for shorebirds flying over the ocean, but this was not the case.

As a result, in the Abstract,

“Using geolocators to track the migration of Icelandic whimbrels (*Numenius phaeopus*), we show that they can complete a round-trip of 11,000 km making two non-stop sea crossings and flying at speeds of up to 24 m s^−1^; the fastest recorded for shorebirds flying over the ocean”.

now reads:

“Using geolocators to track the migration of Icelandic whimbrels (*Numenius phaeopus*), we show that they can complete a round-trip of 11,000 km making two non-stop sea crossings and flying at speeds of up to 24 m s^−1^; one of the fastest recorded for shorebirds flying over the ocean”.

In the Results and Discussion section,

“During the post-nuptial migration in autumn 2012, all four whimbrels flew non-stop to their wintering areas in west Africa (Fig. 2), covering distances of ~3900 to 5500 km in 5 days (Table 1) and, on occasion, achieving the fastest recorded speeds for terrestrial birds on long-distance flight over oceanic waters (up to 18–24 m s^−1^)”.

now reads:

“During the post-nuptial migration in autumn 2012, all four whimbrels flew non-stop to their wintering areas in west Africa (Fig. 2), covering distances of ~3900 to 5500 km in 5 days (Table 1) and, on occasion, achieving some of the fastest recorded speeds for terrestrial birds on long-distance flight over oceanic waters (up to 18–24 m s^−1^)”.

These errors have now been corrected in the PDF and HTML versions of the Article.

