# Supplementary material for: Very rapid long-distance sea crossing by a migratory bird
Source: Sci Rep. 2016 Nov 30;6:38154. doi: 10.1038/srep38154 (PMC5128861; doi:10.1038/srep38154)
Supplement: Supplementary Information [file srep38154-s1.pdf]

Supplementary Information for

"Very rapid long-distance sea crossing by a migratory bird"

Authors

José A. Alves[1,2 *], Maria P. Dias[3,4], Verónica Méndez[5], Borgný Katrínardóttir[6] & Tómas G. Gunnarsson[7]

[1] DBIO & CESAM - Centre for Environmental and Marine Studies, University of Aveiro, Aveiro, Portugal;

[2]South Iceland Research Centre, University of Iceland, Selfoss, Iceland;

[3]Birdife International, The David Attenborough Building, Pembroke Street, Cambridge, CB2 3QZ, UK;

[4]& MARE-Marine and Environmental Sciences Centre, ISPA-Instituto Universitário, Rua Jardim do Tabaco 34, 1149-041 Lisboa, Portugal;

[5]School of Biological Sciences, University of East Anglia, Norwich Research Park, Norwich, NR4 7TJ, UK;

[6]Ecology Department, Icelandic Institute of Natural History, 210 Gardabaer, Iceland;

[7]South Iceland Research Centre, University of Iceland, 800 Selfoss/Gunnarsholt, Iceland.

Correspondence to j.alves@uea.ac.uk

Breeding parameters of Icelandic whimbrels

During the search for nests of tagged Whimbrels in 2013, another 23 first attempt nests were also found for which incubation stage was measured (Liebzeit et al. 2007) allowing laying date to be estimated. All nests were located in the same site, a 2.6 km^2 area of natural river plain area bordered by agricultural land (N 63.788906, W 20.183168).

References

Liebezeit, J. R., P. A. Smith, R. B. Lanctot, H. Schekkerman, I. Tulp, S. J. Kendall, D. M. Tracy, R. J. Rodrigues, H. Meltofte, J. A. Robinson, C. Gratto-Trevor, B. J. Mccaffery, J. Morse, and S. W. Zack. 2007. Assessing the development of shorebird eggs using the flotation method: species-specific and generalized regression models. Condor 109:32–47.

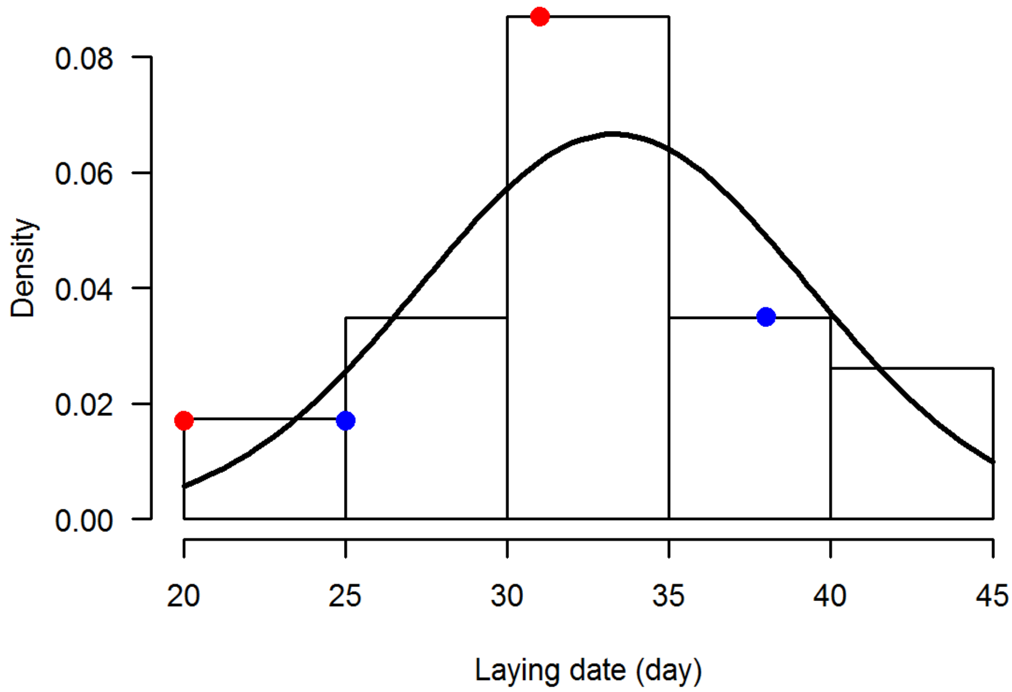

Figure S1. The density distribution of Icelandic whimbrel laying dates (ordinal days since 1st of May), for 23 nests sampled in South Iceland in 2013, showing individuals tracked during non-stop pre-nuptial migration (red) and individuals that made a stop-over (blue).
